# Repair and misrepair of telomeric DNA in dynamic interactions with PML nuclear bodies and lamin B1 in doxorubicin-treated cancer cells

**DOI:** 10.1080/19491034.2026.2688655

**Published:** 2026-07-23

**Authors:** Kristine Salmina, Felikss Rumnieks, Dace Pjanova, Ninel Miriam Vainshelbaum, Jekaterina Erenpreisa

**Affiliations:** a Cell Biology Department, Latvian Biomedical Research and Study Centre, Riga, Latvia; b Institute of Microbiology and Virology, Riga Stradins University, Riga, Latvia

**Keywords:** Cancer treatment, cellular senescence, replication stress, telomere damage, PML nuclear bodies, alternative telomere lengthening, break-induced replication, meiotic proteins, PML fibrillar isoform, lamin B1

## Abstract

Telomeres in epithelial tumors are maintained by telomerase; however, in the MDA-MB-231 breast cancer cell line, treated by doxorubicin (DOX), we found a transient suppression of the telomerase (TERT) before cell growth resumed. Accumulation of cells in late-S-G2/M, mitotic slippage, octaploidy, and decrease of lamin B1 (LMNB1) coincided with this response. The telomere clustering and ALT-like process marked by the telomere shelterin (TRF2) colocalised in PML bodies with DNA DSBs (γH2AX) and recombinase RAD51 were observed in 11–12% of cells. They were preset by arrays of PML-bodies juxta-colocalized with the foci of meiotic prophase proteins SPO11 and DMC1. On the 3rd week, the cells de-polyploidised and returned to the normal cycle, telomerase, and mitosis. ALT-like bodies were also found in BRAFV600E-SK-MEL-28 DOX-treated melanoma cells. However, after sublethal doses of DOX, the formation of PML dimeric rods flanked and tandemly joined by misrepaired TRF2/γH2AX foci occured. Such PML tracts, circumventing cell nuclei undergoing MOS-microtubule-driven rotation, interacted with peripheral chromatin and intermitted with LMNB1 fragments. Furthermore, LMNB1 massively left the nuclear periphery, forming intranuclear flows, and/or convoluted into large peri-nucleolar PML bodies. We interpret our observations as the attempts by damaged, senescing cancer cells to use several mechanisms exploiting PML isoforms and meiotic proteins for telomere repair.

## Introduction

**Figure 1.**
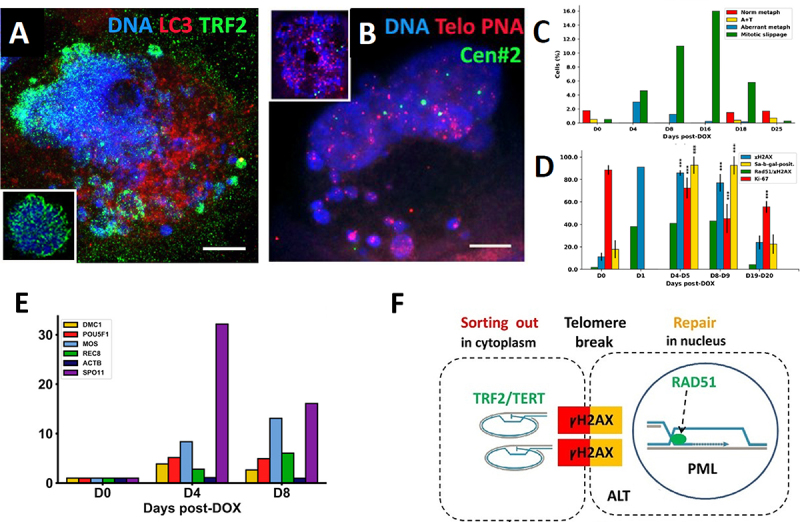
Alternative telomere lengthening (ALT) and repair by homologous recombination in PML bodies (APB) in the polyploidized MDA-MB-231 cells following DOX treatment. (A) Preferential release of the telomere shelterin-TRF2-associated chromatin into the cytoplasm (DOX-D7) (insert: the normal metaphase of a non-treated (NT) cell); (B) Fluorescence in situ hybridization (FISH) with the telomere and cen#2 probes showing retention of centromeres in the cell nucleus and release of a proportion of telomeres into the cytoplasm (DOX-D4) (insert: normal metaphase of NT); (C) representative differential mitotic counts of MDA-MB-231 cells following DOX treatment; (D) the dynamics of the senescence marker Sa-β-gal and proliferation marker Ki-67 along with DNA double-strand breaks (γH2AX) and their repair by homologous recombination—cells with colocalized Rad51/γH2AX foci. (ANOVA with post-hoc analysis (Tukey’s HSD test), *** *p* < 0.001. (E) Expression of the meiotic genes after DOX treatment, shown in folds; (F) a schematic showing the cytoplasmic sorting of hTERT/TRF2-marked DNA damage signalling telomere ends cut off by a telomere break during mitotic slippage. This process is associated with the ALT-RAD51-driven repair by homologous recombination of the two co-aligned trimmed telomeres occurring in specific nuclear PML (APB) bodies. Modified from [15] CC BY 4.0 licence.

Accelerated cell senescence (ACS) imposed by drug treatments on cancer cells paradoxically paves the way to stemness and drug-resistant survival [1–3]. What was once thought to be classic DNA-damaging therapy is now known to entail replication stress (RS) as a major component of ACS and aging [4–6]. For instance, a classic anticancer drug, Doxorubicin (DOX), causes TOPO II inhibition, directly interfering during replication with DNA unwinding and strand separation, and thus induces RS, that is, the stalling of replication forks [7]. ACS is also characterized by telomere attrition [2,3], gradual degradation of lamin B1 (LMNB1) [8–10], activation of transposable elements [11], and transition of TP53 mutants by mitotic slippage (MS) to reversible polyploidy. The cells with reversible polyploidy, in turn, are capable of DNA repair by homologous recombination (HR) of the giant cells resistant to apoptosis [12] and restoration of clonogenic survival of released mitotic descendants [13–15]. Paradoxically, ACS not only accompanies but is even needed for mechanisms based on telomere recombination aided by promyelocytic leukemia (PML) bodies; both mechanisms can coexist [16]. In our previous paper [15], doxorubicin-treated MDA-MB-231 triple-negative breast cancer (MDA-DOX) cells underwent MS, demonstrating senescence and DNA damage in most cells that lasted for two to 3 weeks (reproduced here in Figure 1(A-D) [15]. During polyploidisation following MS, the cells trimmed their telomere overhangs, while also casting off the catalytic unit of telomerase TERT, simultaneously clustering telomeres inside cell nuclei, and transiently undergoing the process similar to alternative telomere lengthening (ALT-like) in PML bodies (APB). Three weeks later, the DNA was repaired, and telomerase-TERT activity was restituted in the clones of the recovered de-polyploidised survivors, resuming mitotic divisions. Figure 1(F) summarizes these findings. Interestingly, in this interim ALT-like period, through the use of RT-PCR, we have observed the expression of several proteins of the meiotic prophase (DMC1, POU5F1, REC8, SPO11), as well as the induction of the meiotic MOS-kinase (Figure 1(E) [15]). Somewhat similarly, a strong dependence of ALT on Hop2-Mnd1, the other two genes used for meiotic homology search, along with directional telomere spinning to another telomere was also previously reported by Cho et al. [17], and a shared mechanism between ALT and the meiotic prophase was proposed [18,19].

The expression of meiotic proteins in somatic tumors, which is abundant, associated with poor prognosis for patients and particularly favored by aneupolyploidy, is long- and well-known [20–28], but the molecular and cellular mechanisms remain unclear and their fidelity disputable. In association with MS or mitotic catastrophe, which is involved in the DNA damage response after various treatments, the expression of meiotic proteins has been a subject of our and colleagues’ studies [29–31]. In some of them, the post-translational component of this meiotic participation in the DNA damage response was revealed for MOS [30] and REC8 [32]. PML nuclear bodies (PML NBs) represent an important multifunctional nuclear organelle involved in post-translational modifications of their multiple ‘clients’ interacting with the inner core proteins inside the PML shell, acting as a ubiquitin ligase, mostly through conjugation with SUMO, and being able to sequester, modify, or degrade partner proteins [33]. Some PML NBs core components (ATRX, DAXX, H3.3, Sirt 1 and others) are responsible for chromatin remodeling [34]. PML is also involved in meiosis [35,36]. Assuming all these data, and taking into account that meiosis starts with the recognition of the chromosome-specific subtelomere homologue sequences [37], we suggested that meiotic prophase proteins, and particularly meiotic recombinase DMC1 (usually presumed to act in pair with RAD51) and meiotic recombination nuclease SPO11, may be involved in the repair of telomeres during the transient ALT-like process [15].

Furthermore, considering the close ties of mammalian meiotic prophase telomeres with the nuclear envelope (NE), their attachment through telomere-capping shelterins TRF1/2 to lamin B1, and their moving along the NE in the meiotic prophase [38,39], in sum with the data on the antagonistic relationship between the telomere-NE binding and ALT [40], we were also interested in the topological relationship of ALT components with Lamin B1 (LMNB1) and peripheral heterochromatin. In a wider context, the topological relationship between LMNB1 and heterochromatin for determining genome stability in DNA damage and even in SHM [41,42] presents a subject of high interest and ongoing studies [43].

Therefore, in the current study, we reproduced the MDA-MB-231-DOX experiments and added to our immunofluorescence set the antibodies for SPO11 and DMC1, MOS, and LMNB1 (see Table 1 in Methods). To extend our study, we also performed experiments on the melanoma cell line SK-MEL-28 (BRAFV600E- and CDK4,6-mutant) [44] and applied mild and sublethal concentrations of 24-h doxorubicin treatment (SK-MEL-DOX). Both cell lines, triple-negative breast cancer and BRAF-mutant melanoma, represent some of the deadliest human cancers. These cell lines are similar, both also being TP53 mutant, metastatic and aneuploid (para-triploid and para-tetraploid, correspondingly), and thus prone to replication stress [45]. Therefore, their adaptations to anticancer drugs via PML NBs with meiotic proteins are of interest. As shown below in the Results, we have observed the association of SPO11 and DMC1 with PML arrays presetting mature ALT-like yH2AX/RAD51 PML bodies, after DOX-treatment of both cell lines, at the mild DOX concentration of 100 nM. However, at sublethal DOX (250–500 nM) concentrations, we unexpectedly found the formation of fibrillar PML structures (apparently, the PML II isoform known for senescence [46,47] and for its affinity to the nuclear envelope [48]), also described in laminopathy progeria [49]). Therefore, we investigated additionally by confocal microscopy the topological relationship between telomere/TRF2-enclosing fibrillar PML structures and the nuclear periphery, and the relationship with RAD51-dependent homologous recombination (HR) in their dynamic interaction with LNMB1 and chromatin in the DOX-treated cells. We interpret our observations as the attempts to use several PML isoform-related mechanisms to recover the badly damaged aneuploid cancer cells, at the brink of catastrophic breakdown of the nuclear integrity.

**Table 1.** The antibodies, their specificity, and their source.

Antibody against	Description	Specificity/Immunogen	Used concentration	Product no. and manufacturer
α-Tubulin	Mouse monoclonal	Recognises an epitope located at the C-terminal end of the α-TUBULIN isoform in a variety of organisms	1:1000	T5168, Sigma-Aldrich, St. Louis, MO, USA
DMC1	Rabbit polyclonal	Recombinant fragment corresponding to a region within amino acids 1 and 206 of DMC1	1:100	PA5-21472, Thermo Fisher Scientific, Waltham, MA, USA
γ-H2AX	Rabbit polyclonal	Recognises mammalian, *yeast Drosophila melanogaster* and *Xenopus laevis* γ-H2AX	1:200	4411-PC-100, Trevigen, Gaithersburg, MD, USA
γ-H2AX	Mouse monoclonal	Synthetic peptide sequence surrounding phosphorylated Ser140	1:200	MA1-2022, Pierce, Waltham, MA, USA
LAMIN B1	Rabbit polyclonal	Peptide mapping at the C-terminus of Lamin B1 of human origin	1:200	ab1604, Abcam, Cambridge, UK
LC3	Rabbit polyclonal	The details of the immunogen for this antibody are not available	1:100	ab63817, Abcam, Cambridge, UK
MOS (C237)	Rabbit polyclonal	Epitope mapping at the C-terminus	1:50	sc-86, Santa Cruz, Dallas, TX, USA
PML	Rabbit polyclonal	A synthetic peptide corresponding to the N-terminus of the Human PML/RNF71/TRIM19	1:200	PA5-80910, Thermo Fisher Scientific, Waltham, MA, USA
PML	Mouse monoclonal	Epitope corresponding to amino acids 37–51 mapping near the N-terminal of PML of human origin	1:200	sc-966, Santa Cruz, Dallas, TX, USA
RAD51	Mouse monoclonal	Recombinant full-length protein corresponding to Human Rad51 aa 1–338	1:50	ab213, Abcam, Cambridge, UK
SPO11	Mouse monoclonal	raised against amino acids 97–396 mapping at the C-terminus of Spo11 of human origin	1:50	sc-377161, Santa Cruz, Dallas, TX, USA
TERT	Mouse monoclonal	Recombinant full-length protein (human) from insect cells	1:50	ab5181, Abcam, Cambridge, UK
TRF2	Rabbit polyclonal	Recombinant protein corresponding to Human TERF2. Recombinant protein control fragment (Product #RP-101881< /a>).	1:100	PA5-81990, Thermo Fisher Scientific, Waltham, MA, USA
TRF2	Mouse monoclonal	His-tagged, fusion-protein, corresponding to full-length TRF2 (Telomeric Repeat binding Factor 2)	1:100	05–521, Millipore, CA, USA Temecula,

## Materials and Methods

### Cell lines and treatment

The triple-negative para-triploid breast adenocarcinoma MDA-MB-231 cell line was obtained from the ECACC (Cat. No. 92020424, European Collection of Authentic Cell Cultures, Wiltshire, UK). The para-tetraploid human melanoma SK-MEL-28 cell line (mtB-RAF V600E, mtTP53) was obtained from the ATCC (Cat. No. HTB-72, The American Type Culture Collection, Manassas, VA, USA). Cells were cultured in flasks in Dulbecco’s modified Eagle’s medium (DMEM, Cat. No. D6429, Sigma-Aldrich, St. Louis, MO, USA) supplemented with 10% fetal bovine serum (FBS; Cat. No. F7524, Sigma-Aldrich, St. Louis, MO, USA) at 37°C in a 5% CO2 humidified incubator without antibiotics. For experimental studies, cells were maintained in the log phase of growth and treated at 60–80% confluence with DOX (doxorubicin, Cat. No. D1515, Sigma-Aldrich, St. Louis, MO, USA) for 24 h at several concentrations (100, 250, 500, 1000 nM) for SK-MEL-28 cells and 100 nM for MDA-MB-231 cells. After drug removal, the cells were maintained by replenishing the culture medium every 2–3 days and sampled over 3 weeks post-treatment until the appearance of escape clones. In some experiments, cells were grown on chamber slides (Cat. No. 154526, Thermo Fisher Scientific, Waltham, MA, USA).

To determine growth kinetics, cells were seeded and treated with DOX at a density of 100,000 cells per well in a 6-well plate, and counts were performed using a Neubauer camera (Heinz Herenz Medizinalbedarf GmbH Hamburg, Germany) and staining with 500 µl Trypan blue dye (0.4%, Cat. No. T8154, Sigma-Aldrich, St. Louis, MO, USA) added to 500 µl of cell suspension.

### Immunofluorescence

Immunofluorescence staining was performed as previously described [15]. Briefly, cells were pelleted, suspended in 100 µl warm FBS, and pelleted by cytocentrifuge again onto polylysine-coated glass slides or grown and fixed on chamber slides. They were fixed in 50 ml methanol for 7 min at–20 °C, dipped 10 times in 50 ml ice-cold acetone, and allowed to briefly dry. Slides were then washed three times in 50 ml Tris-buffered saline (TBS) 0.01% Tween 20 (TBST) for 5 min and blocked for 15 min in 100 µl TBS, 0.05% Tween 20, and 1% bovine serum albumin (BSA) at room temperature. Samples were covered with 50 µl TBS, 0.025% Tween 20, and 1% BSA containing the primary antibody and incubated overnight at 4 °C in a humidified chamber. The primary antibodies and their sources are listed in Table 1. Samples were then washed three times in 50 ml TBST and covered with 100 µl TBST containing the appropriate secondary antibodies 6,7 µg/mL (goat anti-mouse IgG Alexa Fluor 488 (A11001 Invitrogen, Carlsbad, CA, USA) and goat anti-rabbit IgG Alexa Fluor 594 (A11037, Invitrogen, Carlsbad, CA, USA) and incubated for 40 min at room temperature in the dark. Slides were washed three times for 5 min with 50 ml TBST and once for 2 min in PBS, counterstained with DAPI (0.25 µg/mL) and finally embedded in Prolong Gold (Invitrogen, Carlsbad, CA, USA). For TERT staining, DNA denaturation was performed using 50 ml 2 N HCl at 37°C for 20 min before the blocking step. When staining for α-TUBULIN, the post-fixation drying step was omitted and fixation in 50 ml 4% paraformaldehyde for 15 min was performed. For PML detection two different primary antibodies (monoclonal and polyclonal, see details in Table 1), which fully match each other (Figure S1) were used, to be able to combine them with other antibodies of interest. The validity of DMC1 and SPO11 antibodies was checked in adult Wistar rat spermatocytes (Figure S2). The rats were previously explored in the experiment approved by the Committee of Bioethics at R.E. Kavetsky Institute of Experimental Pathology, Oncology and Radiobiology (protocol № 1a of 16 February 2017) and were obtained for reuse. The Mos antibody was checked by Western blotting earlier [15].

For microscopy, a fluorescence light microscope (Leitz Ergolux L03-10, Leica, Wetzlar, Germany) equipped with a color video camera (Sony DXC 390P, Sony, Tokyo, Japan) was used. Confocal microscopy was performed using a Leica TCS SP8 laser scanning confocal microscope (Leica Microsystems, Wetzlar, Germany). Four laser lines were used: diode (405 nm) for DAPI, argon (488 nm) for AlexaFluor488, and DPSS (561 nm) for AlexaFluor594. Z-stacks and focal planes were scanned using 63×/N.A. 1.40 objective. Images and Z-stack maximum projections were then processed and analyzed using Leica Application Suite X software (Leica Microsystems, Germany). Three-dimensional Z-stack reconstruction was performed using LAS X 3-D Viewer (Leica Microsystems, Germany). To capture epifluorescence images, in addition to separate optical filters, a three-band BRG (blue, red, green) optical filter (Leica, Wetzlar, Germany) was applied.

### Toluidine blue DNA staining and image cytometry

Cytospins were prepared and fixed with 50 ml ethanol/acetone (1:1) for >30 min at 4°C and air-dried. Slides were then hydrolyzed with 50 ml 5 N HCl for 20 min at room temperature, washed in 50 ml distilled water (5 × 1 min) and stained for 10 min with 500 µl 0.05% toluidine blue in 50% citrate-phosphate McIlvain buffer at pH 4. Slides were rinsed with 50 ml of distilled water, blotted dry, and dehydrated by incubating twice in 50 ml butanol for 3 min each at 37°C. Samples were then incubated twice in 50 ml xylene for 3 min each at room temperature before being embedded in DPX. Digital images were collected using a Sony DXC 390P color video camera (Sony, Tokyo, Japan) calibrated in the green channel. DNA content was measured as the integral optical density (IOD), using Image-Pro Plus 4.1 software (Media Cybernetics, Rockville, MD, USA). Arbitrary 2C DNA values were averaged from measuring anaphases in untreated tumor cells. The method error was estimated to be less than 10%. For cell cycle measurements, 200–500 interphase cells were collected at each point.

### Fluorescence in situ hybridization (FISH)

Telomere FISH for Telo PNA Cy3/Cen#2 FITC was performed with a peptide nucleic acid (PNA) telomere probe (Dako Inc., Glostrup, Denmark) in conjunction with a differentially colored centromere 2 PNA probe (a gift from Dako, Inc., Glostrup, Denmark) as an internal reference point. FISH was applied following the procedure previously described [15,50].

### Re-analysis of a previously established MDA-MB-231 transcriptomics dataset

A dataset of MDA-MB-231 transcriptomes (comprised of three replicates each for five conditions – non-treated/NT and days 5, 8, 16 and 22 post-DOX (D5, D8, D16, D22) that has been previously sequenced and analyzed [51] was investigated further by adding extra comparisons of differential gene expression analysis between time-points (namely, D8 vs D5, D16 vs D5, D16 vs D8, D22 vs D16, D22 vs D5). DE analysis was performed via the same procedure as [51], with the gene-level count matrix (obtained using tximport [52]) and sample annotation used as input to edgeR [53] to obtain differentially expressed genes (DEGs) with the glmQLFTest approach. The threshold for differential expression was selected to be FDR <0.05 and LogFC >1 (in absolute value). The expression dynamics of selected genes and transcripts of interest were visualized using the ggplot2 package [54]. The list of DEGs for all timepoint comparisons is enclosed in the supplementary materials (File S3). The dataset was submitted to ArrayExpress and assigned the E-MTAB-16509 accession number.

## Results

### DOX-treated cell lines growth curves and cell cycle with DNA cytometry

**Figure 2.**
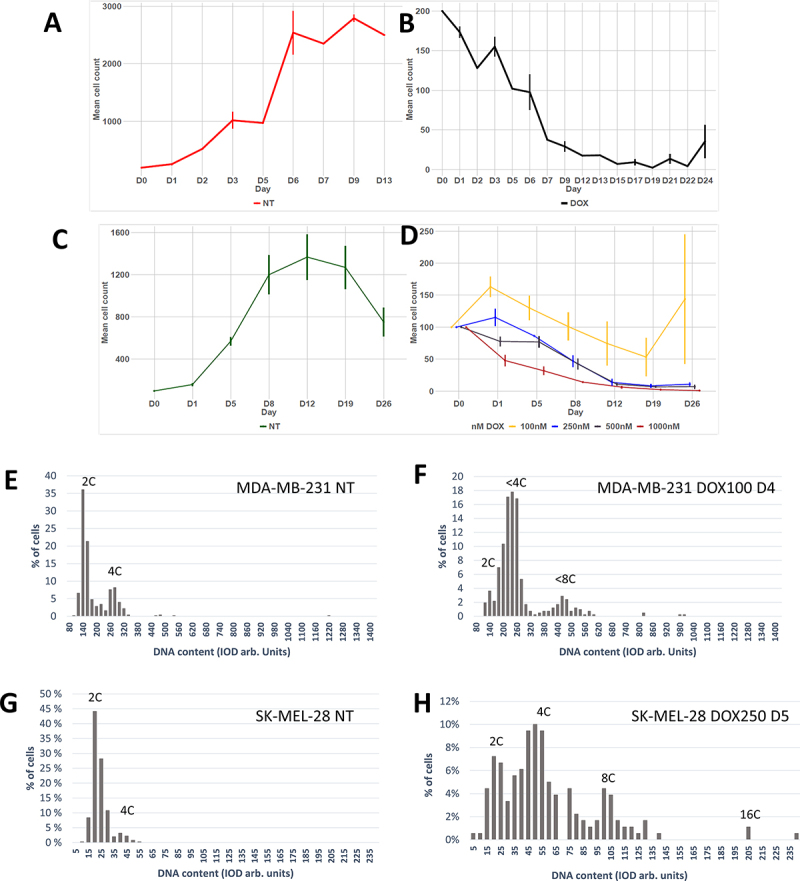
Cell growth and cycle. (A,B) the MDA-MB-231 cell growth curves for three independent experiments of non-treated (NT) cells (A) and after DOX treatment (B) (with SE); (C,D) the SK-MEL-28 cell growth curves for three independent experiments of NT cells (C) and after DOX treatment (D)(with SE); (E-H) typical DNA cytometry histograms showing DOX treatment induced delay in late S-G2 phase of the cell cycle, polyploidisation to 4C and 8C fraction at days 4–5 (standard error = 5.68%, *n* = 180): (E) MDA-MB-231 NT cells; (F) MDA-MB-231 DOX cells on day 4 after treatment; (G) SK-MEL-28 NT cells; (H) SK-MEL-28-DOX cells on day 5.

As can be seen, on Figure 2(A,B), MDA-MB-231 cells treated with one dosage of DOX100nM steeply decrease cell growth from day 5 to 9, keep low cell numbers till the end of the 3^rd^ week, and then resume cell growth from day 23–24; Figure 2(C,D), showing response to the mild and higher DOX dosages on SK-MEL-28, somewhat similarly first reveal descending cell growth from day 1, which at sublethal dosages (DOX 250–500 nM) becomes steep from day 5. At mild DOX100 cells resume growth from day 19, but after sublethal DOX dosages they lag at low cell numbers and slowly increase cell growth by day 26. At the lethal dosage of DOX1000nM, the cell number is significantly dropped. Already from day 1, it continues to slowly decrease to near zero numbers till the end of the experiment. These cells do not survive. It can be noted that in both cell lines, we observed a steep drop of cell growth from day 5 for a week or less; at that period we found the higher marking of senescence, DNA DSBs, colocalised with RAD51, and a noticeable increase of mitotic slippage, which could favor endoreplication and replication stress (RS); hence, most of these cells were concomitantly expressing Ki-67 (Figure 1(C,D)).

Indeed, a considerable fraction of cells in both tumors delayed in late S-G2M (4C) and doubled its DNA content on days 4–5 (8C) as seen by DNA image cytometry (Figure 2(E-H)). In the case of SK-MEL-28–250 nM, there was a more pronounced accumulation of the 8C fraction, as well as the start of the next doubling (16C). As shown previously, after reaching 8C-16C polyploidy on days 16–18, the cells started a notable return route to 2C cell cycle recovery [15].

### The topological relationship between the ALT components and lamin B1 over time post-DOX action. DNA repair and misrepair

Non-treated (NT) breast cancer MDA-MB-231 and melanoma SK-MEL-28 cell lines in immunofluorescent staining with the used antibodies express some amount of meiotic recombination nuclease SPO11 and meiotic recombinase DMC1 particles; the cells are also MOS-kinase positive, particularly polyploid as reported earlier [15,55].

The spatial interactions between the marker of telomere end shelterin TRF2, the PML protein, the marker of DNA double-strand breaks γH2AX, the SPO11, RAD51, and DMC1 recombination-related proteins, and LMNB1 structures were examined by confocal microscopy in non-treated (NT) cells and cells after 24 h-DOX treatment on days 4–9 (when most cells delay in late S-G2M-phase, enter MS and polyploidise (Figure 2 and [15]). In general, the topology and interaction of these nuclear components in response to DOX can be divided into five phases.

#### Phase I-II (provisional orientation of ALT components)

**Figure 3.**
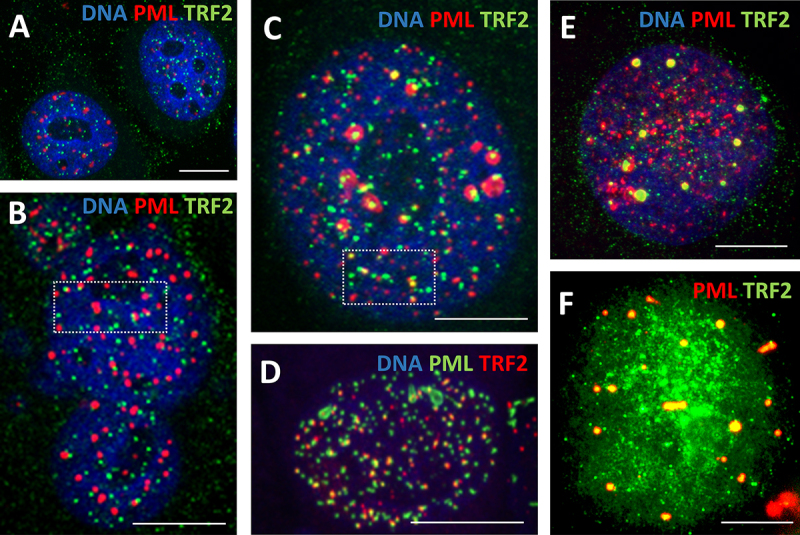
The topology and interaction of ALT-like process components (PML bodies and TRF2) in response to DOX. (A) NT SK-MEL-28 and (B) DOX 250 nM-D5 cell showing co-parallel orientation of the long sparse arrays of the small PML pre-bodies (0.2 < 1 µm) and tiny TRF2 foci; (C) SK-MEL-28 cell with mature ~1–2 µm round APB-like bodies including TRF2 foci; insert shows short arrays of more apposed and increasingly colocalising components of maturing APB-like nuclear bodies (DOX250nM-D5); (D) another pair of PML/TRF2 antibodies (see Table 1 in Methods) also shows a similar juxta-position, short chains and frequent colocalisation of both components in SK-MEL-28 cell (DOX250nM-D5); (E) SK-MEL-28 cell (DOX250nM-D5) and (F) MDA-MB-231 cell (DOX100nM-D7) both showing mature APB-like NBs colocalising with TRF2 foci. Bars = 10 µm.

Phase I: Co-parallel orientation of the long, sparse arrays of the small fissed PML-pre-bodies and tiny TRF2 loci, with their juxtaposition and occasional colocalisation (approximately one in ten foci), was seen in 42 ± 4% of non-treated (NT) melanoma and breast cancer cells, 45 ± 5% in SK-MEL-DOX and 53 ± 1% in MDA-DOX cells, on day 4–5 after 24 h treatment (evaluated in two independent experiments). The described pattern for NT SK-MEL 28 cells is presented in Figure 3(A). Although in a number of co-parallel orientations of the mentioned components, the NT cells were rather similar to DOX-treated cells, it was more typical for the multilobulated large cell nuclei (Figure 3(B)). The orientation of small SPO11 foci and DMC1 was more apparent in the abundant Phase II, the further development of the process.

**Figure 4.**
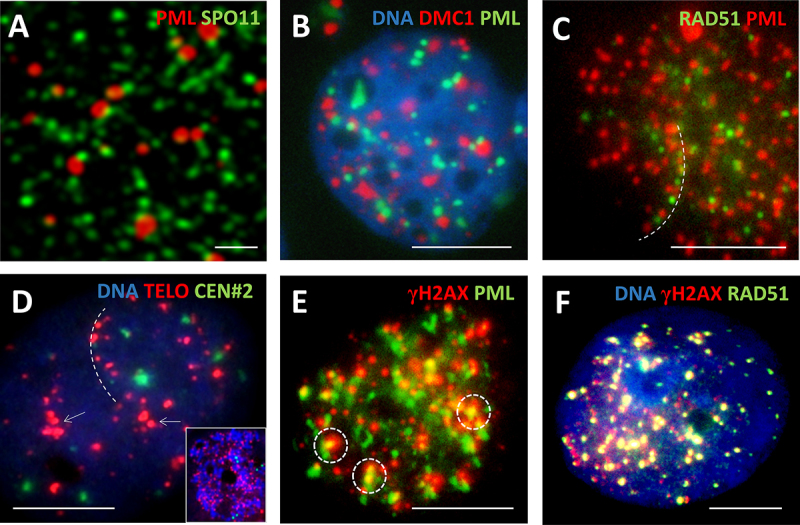
Interaction and colocalisation of the DNA recombination proteins with maturing ALT-like structures in response to DOX. (A) Interaction of PML bodies with the arrayed SPO11 foci in SK-MEL-28 cell, arrowed (DOX250nM-D5); (B) SK-MEL-28 cell showing interaction of the arrayed PML pre-bodies with DMC1 recombinase (DOX250nM-D5); (C) Tandem alignment of PML and RAD51 positive repair foci in SK-MEL-28 cell, the colocalisation spot is arrowed (DOX250nM-D5); (D) Fluorescence in Situ Hybridization with the telomere and cen#2 probes showing arrays (dashed line) and clusters (arrows) of telomere DNA sequence in SK-MEL-28 cell (DOX500nM-D5); insertion - prometaphase in the untreated cell showing red labels on the chromosome ends; (E) Formation of mature APB-like bodies including γH2AX foci in MDA-MB-231 cell (DOX100nM-D5); (F) Colocalization of γH2AX and RAD51 repair foci in MDA-MB-231 cell (DOX100nM-D5). Bars = 10 µm.

Phase II (Figure 3(C) (boxed) and Figure 4(A-C)): short tandem arrays of more closely located and increasingly juxtaposed/colocalising components of the PML-associated, ALT-like process (approximately one in four foci), including PML, TRF2, γH2AX with RAD51, some of which terminated in maturing nuclear bodies (APB), were observed. PML and TRF2 foci colocalisation in both cell lines was confirmed by two different pairs of antibodies (Figure 3(C-F)), listed in Methods/ Table 1. Although SPO11 was found in some APB bodies, it did not show direct colocalization with TRF2 protein (not shown).

The arrays and clusters of telomeres in cells treated with DOX were also confirmed by Telo-FISH (Figure 4(D); for the control metaphase, see the insert). Short tandem arrays are also described in the literature as possibly caused by the formation of the ubiquitinated substrates for the formation of functional APB [56].

Our observations on the orientation of TRF2 and meiotic components with PML pre-bodies preceding the maturation of APB bodies indicate that it is likely associated with the late S-phase. The S-phase is known to attract fissed PML pre-bodies that are undergoing fission-fusion events, particularly enhanced by the RS that is typical for aneuploid tumors [57,58]. RS was clearly enhanced after DOX, as demonstrated by S-phase delays on Figure 2(F-H). Furthermore, we can consider the ALT-like break-induced replication (BIR), which is a kind of recombination resolving RS and also needing PML NBs, which mediate the recruitment and release of the recombination intermediates of telomere repair for both ALT or BIR [59]. Our observations are also in line with the data that ALT is a self-organizing process in ALT-associated PMLNBs [33,60].

Coinciding with our observations, the strand composition of these recombination intermediates was previously described as an indicator of the constrainment of these processes to a narrow time-space window in the cell cycle following replication [61].

#### Phase III (presumed maturation of recombinogenic APBs)

It is known that mature APBs harbor actively replicating telomeres and the telomere repeat binding sumoylated TRF1/TRF2 factors in the late S-G2 phase of the cell cycle [33]. In our samples, these short arrays appeared forming mature ~1–3 µm round APB-like bodies including TRF2 foci, γH2AX, and RAD51 as found in 11–12% of cells, mostly 4~8C (as judged by nuclei size and density in DAPI staining), on days 4–9 post-DOX (Figure 3(C-E)) and Figure 4(E-F)).

#### Phase IV: the formation of PML II isoform fibrillar structures

**Figure 5.**
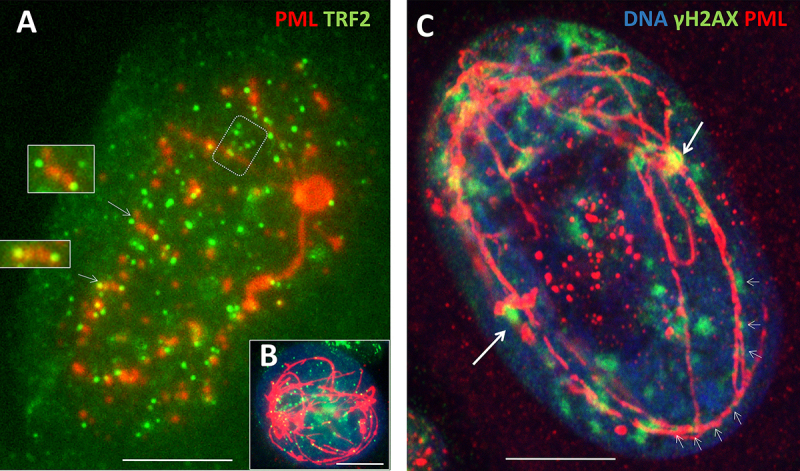
Formation of PML II isoform fibrillar structures following SK-MEL-DOX treatment. (A) The cell showing long PML tracks assembling from dimeric rods of PML NB flanked by TRF2-positive foci (arrows and on insert), DOX500nM-D5; (B) PML tangle with TRF2 foci resembling the meiotic zygotene bouquet, DOX500nM-D5; (C) dimeric PML segments dashed by γH2AX links (small arrows) that are indicative of the non-homologous end-joining of DNA double-strand break-containing telomere repeats sticking in the PML tracts; long strands of PML aggregate and colocalise and form bows and tangles around large H2AX-positive chromatin clusters (big arrows), (DOX250nM-D5). Bars = 10 µm.

The PML gene is composed of nine exons and has six nuclear isoforms (with mutual 1–3 exons). Only one of them, PML II with a highly disordered Exon 7b, forms the fibrillar structures with affinity to the nuclear envelope (NE) [48]. After sublethal doses 250 nM and 500 nM DOX, PML fibrils constituting long ribbons of looped structures were observed, much more frequently in melanoma than in MDA-DOX cells. It appears that the long PML tracts were arrayed by dimeric PML rods flanked by TRF2-positive foci (Figure 5(A)). Occasionally, fibrillar structures strikingly reminiscent of meiotic zygotene ‘bouquets’ attracted to the NE were observed (Figure 5(B)). PML dimeric segments were concurrently dashed on each side by γH2AX/DNA insertions (Figure 5(C), small arrows). These long tracts of PML tended to circumvent the nuclear periphery and formed tangles (Figure 5(A)) and bows around and within large clusters of the γH2AX-positive chromatin (Figure 5(C), big arrows).

#### Fibrillar PML strands are largely void of RAD51 foci in DOX-treated cells

**Figure 6.**
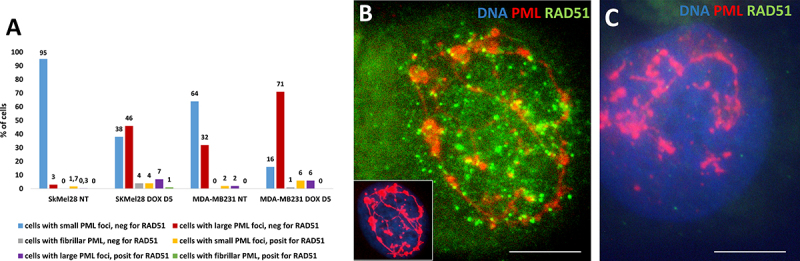
Fibrillar PML as related to RAD51-labelled DNA recombination repair after DOX: (A) The counts of cells containing small, large round and fibrillar structures of PML for the presence of RAD51 foci after DOX treatment (250 nM for SK-MEL-28 and 100 nM for MDA-MB-231 cells) on day 5; (B) Fibrillar PML structures with RAD51 foci in SK-MEL-28 cell and PML-DAPI, in insert (DOX250nM-D5); (C) Fibrillar PML structures negative for RAD51 in SK-MEL-28 cell (DOX250nM-D5). Bars = 10 µm.

During meiosis, recombinase RAD51 forms discrete nuclear foci on synaptonemal complexes from early zygotene to late pachytene [62]. We wondered if and how somewhat similar fibrillar PML structures may be involved in the recombinational DNA repair, and inspected them for the presence of RAD51 foci after using DOX for both cell lines in comparison with cells displaying the ALT-like foci; the counts are presented in Figure 6(A) for day 5. They show that the proportion of such RAD51-positive cells with fibrillary PML structures was found in only 1% in SK-MEL-DOX (250 nM), while RAD51-negative PML fibrillar structures accounted for 4%. The proportion of RAD51-positive cells with small and large APB-like bodies comprised a sum of 11%. MDA-DOX (100 nM) similarly showed 12% cells with RAD51-positive APBs and only 1% cells with extensive PML fibrillar structures lacked RAD51. Therefore, in general, the conversion of the ALT-like PML foci into fibrillar PML forms is associated with drug toxicity and, likely, the inability to repair telomeres by homologous recombination (HR). Nevertheless, melanoma cells, relatively more proficient in creating fibrillar PML at sublethal DOX (250–500 nM) concentrations, still (although barely) survived, as seen from the growth curves in three independent experiments (Figure 2(D)). As for the topology in these rare cells with RAD51-positive fibrillar structures, the pattern of RAD51 foci is rather chaotic (Figure 6(B)). A similar, more frequent and unlabeled-for-RAD51 PML fibrillar pattern is shown in Figure 6(C).

The factors limiting the recombinative telomere repair in the PML II structures may be due to the lack of the essential PML body inner core proteins, SP-100, SUMO, and DAXX [34]. Their absence was reported in the case of the PML II fibrillar isoform when transfected [48] or as transient structures in differentiation-induced human embryonic stem cells [63]. Along with the loss of the inner core of PML NBs, the affinity of the fibrillar PML structures to the nuclear periphery and nuclear lamina was also reported in both cited papers. Here, it should be noted that the gradual degradation of lamin B1 (LMNB1) accompanies the ACS [8–10], while its experimental deletion causes the spinning of cell nuclei [64]. We confirmed here both the reduction of lamin B1 (LMNB1) and nuclear spinning.

#### LMN1 and TERT are transiently suppressed in the dynamics of post-DOX treatment

**Figure 7.**
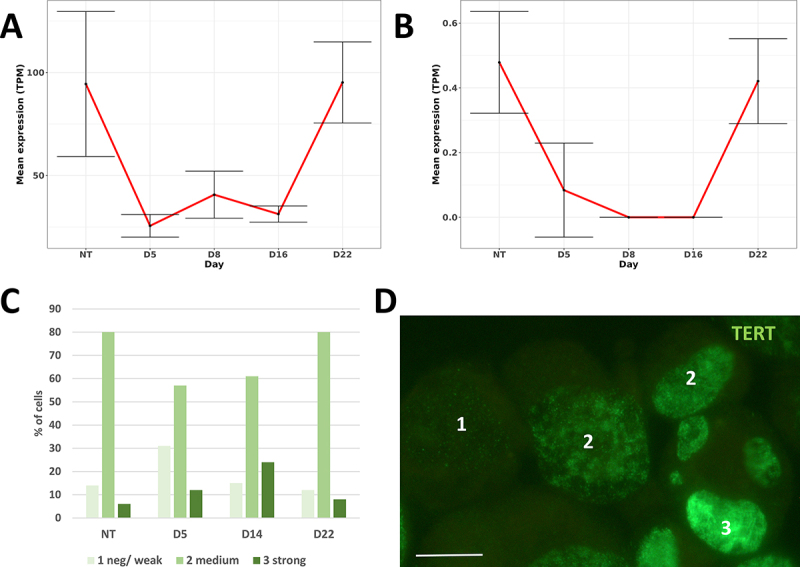
LMNB1 and TERT expression after DOX treatment in MDA-MB-231 cells. (A) the expression of LMNB1 in a time-series transcriptomics dataset; (B) the expression of TERT in a time-series transcriptomics dataset; (C) changes of TERT-immunostaining intensity by semi-quantitative counts; (D) an example of semi-quantitative evaluation of TERT-protein immunostaining intensity in cell nuclei. Bars = 10 µm.

To assess the level of telomerase and LMNB1 in their relationship to the ALT-like process, their level of expression (both general and differential) was assessed in a time-series dataset of 15 non-treated and DOX-treated MDA-MB-231 transcriptomes (3 replicates each for non-treated/NT and days 5, 8, 16 and 22 post-DOX) that had previously been sequenced and analyzed by Salmina et al. (2023) [51]. Multiple timepoint comparisons demonstrated differential expression of TERT, with it being statistically significantly downregulated in D5, D8 and D16 compared to NT, but upregulated in D22 compared to D16. TERT and LMNB1 expressions over time are visualized in Figures 7(A,B) and show a similar downregulation on days 5–16. In accord, using immunofluorescence staining, we found changes in TERT-protein expression intensity. As shown in Figures 7(C,D), DOX treatment increased the number of cells with low and abnormally high levels of TERT (with the impaired degradation) and decreased medium staining at the same time period, as compared with non-treated cells. On D22 TERT expression returned to NT levels.

#### Interaction of PML with the nuclear envelope and rotation of DOX-treated cell nuclei

**Figure 8.**
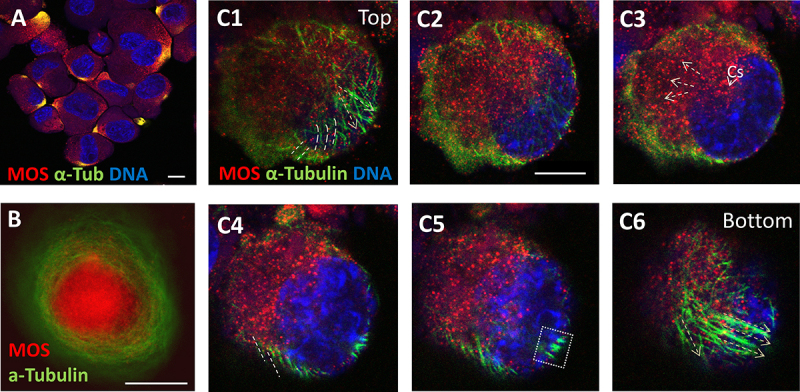
Rotation of MDA-MB-231 breast cancer cell nuclei immunostained for MOS and α-TUBULIN and counterstained with DAPI: (A) Control cells without treatment; (B-C) MDA-MB-231 cells after 100 nM Doxorubicin treatment (day 4) displaying on (B) the concentric rings of microtubules, stained by α-TUBULIN, around MOS-enriched cell nucleus; (C1-6) Confocal sections from stack-images through one prophase-like (~4C) cell showing both MOS- and chromatin-associated α-TUBULIN (C5 frame), with chiral symmetry break (C1: clockwise and C6: anticlockwise). The directed attachment of MOS particles emanating from a centrosome (CS on C3) and the microtubules attached to them are dash-labelled and to DNA, enframed (C5). Bars = 10 µm.

The latest review [40] indicates that the redistribution of telomeres from their intranuclear localization to the nuclear envelope (NE), whether artificial or in senescence, prevents APB formation and likely reduces ALT. This shift requires the attachment of telomeres to SUN-proteins of the inner NE, which are linked to the cytoskeleton through the interaction of KASH proteins of the outer NE. Interestingly, the same occurs with the meiotic prophase. For that, the meiotic prophase needs some flexibility of LMNB1 [38,40]. The observed redistribution of the PML tracts circumventing the nuclear periphery may be similarly associated with nuclear rotation, due to the partial LMNB1 degradation (shown on Figure 7(A)), which at the same time serves as a shelter for telomeres [8–10,40]. We checked for nuclear rotation in MDA-DOX cells (senescent by 80% as shown earlier [15]). For that, we used confocal microscopy of cells stained for DNA, α-TUBULIN and the meiotic kinase MOS, which is known for its capacity to phosphorylate microtubules and by its likely organization of the meiotic prophase with a monopolar spindle upon mitotic slippage [15,65]. We found the involvement of microtubules attached to the meiotic kinase MOS in the rotating cell nuclei. The rotation of DOX-treated cell nuclei, observed in this experiment indirectly, is presented in Figure 8.

In Figure 8(A), the untreated cells are presented with MOS localization at the interphase centrosome. MOS binding with centrosomes has been found in many TP53-mutant cancers [66]. In Figure 8(B), the MDA-DOX cell nucleus and perinuclear area are highly enriched with MOS surrounded by concentric rings of microtubules. Enrichment with MOS was previously confirmed in these cells by RT-PCR and Western blotting [15]. On Panel C1–C6 the confocal stack-images through one MDA-DOX treated cell on day 4, possessing the features of the prophase, are presented. The spatial arrangement of the microtubule fibrils causes chiral symmetry breaking (compare C1 with C4-C6), which is indicative of nucleus rotation (thanks to Kenichi Yoshikawa for a personal comment). The MOS-kinase particles circumventing and emanating from a centrosome (C3, Cs label) show directed attachment to both MTs (C4, dash-labeled) and chromosomes (C5, enframed) that likely serves as a driving force of this rotation.

#### Topological relationship of PML tracts with lamin B1 (LMNB1), chromatin, and nucleoli (examined in SK-MEL-DOX cells)

**Figure 9.**
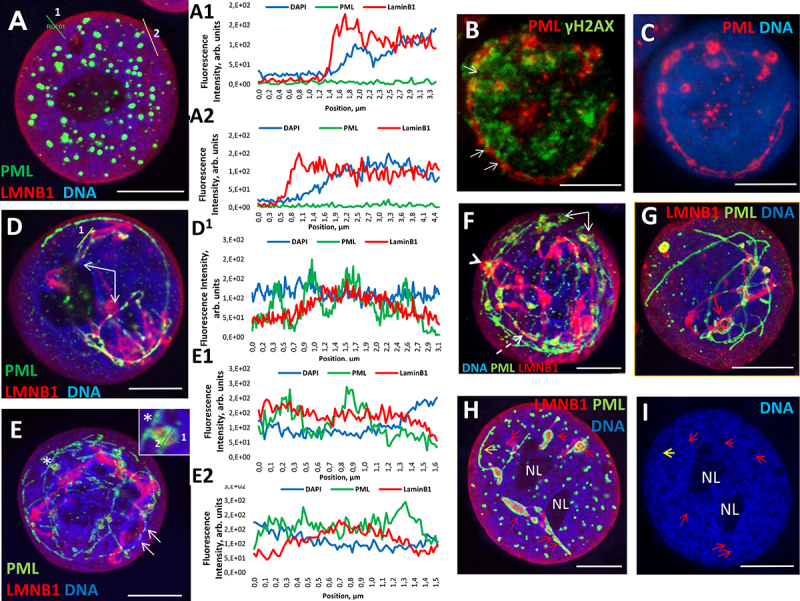
PML fibrillar structures in their topological relationship with lamin B1 (LMNB1), chromatin, and nucleoli (SK-MEL-DOX (250 nM, Day 5), confocal images: (A) a non-treated cell nucleus displays conventional PML-NBs; one-line densitograms through the nuclear envelope (1 and 2) are presented on the panels A^1^ and A^2^ showing tight link between LMNB1, and the chromatin; (B) PML tracts with small loops make two concentric rings around nuclear periphery in the cell, γH2AX-positive chromatin insert between fibrillar PML segments (arrows) and is continued inside cell nuclei; (C) the same cell as in fig. B, PML, DAPI channels: small loops in circumventing PML tracts are well seen; (D, E) PML fibrillar segments circumvent the nuclear periphery, mostly colocalise with chromatin (arrows on E) and form small “curls,” often intertwisted with LMNB1 (asterix and insert on E, and double-arrow on F) and in its inner branches. Panels E^1^ and E^2^ show two perpendicular intensitograms showing twisting across the asterisk-labelled structure with a twist between PML and LMNB1 but with the chromatin emptied inside; in Figure 9(F) the PML spot entrapped in LMNB1 structure is arrowhead and a segment of the tight colocalization of PML and LMNB1 is dash-arrowed; on Figure 9(F) the concentric rings of PML tracts are preserved, but much of LMNB1 develops shapeless intranuclear flows and forms holes in the chromatin (red arrows on (G-I). At this stage, the PML tracts circumventing the nuclear periphery are absent while LMNB1 supra-structures enrol near the nucleoli (NL) into the large PML-NBs. Red arrows indicate the sites of LMNB1 convolutes into large PML-NBs which correspond to the holes in the chromatin (well seen in (I), by the yellow arrow on (H and I) the remnant of the circular PML, also void of the DAPI staining. The double-red arrow on (H-I) indicates the channel towards the NE. Bars = 10 µm.

In non-treated control SK-MEL-28 cells, the PML bodies typically display intranuclear localization (see also Figure 3(A)) and are separated from LMNB1, which interacts with the aligned peripheral heterochromatin (Figure 9(A) and line scans A^1^ and A^2^).

However, after sublethal DOX treatment (250 nM, day 5), long PML tracts with small curls were often seen as two concentric rings circumventing the nuclear periphery, presuming rotation around it. These nuclei are rich with the γH2AX-positive chromatin joined to the peripheral chromatin through the dashed gaps in concentric PML tracts (Figures 5(C) and 9(B), small arrows, and 9C for DAPI/PML). Furthermore, we observed that PML tracts became partially displaced from the nuclear periphery, forming intranuclear loops of PML intermitted with LMNB1 dashes (Figure 9(D)) along with thick LMNB1 flows of uncertain configuration inside nuclei (Figure 9(D–F)). However, at this stage of senescence, the fibrillar PML mostly still preserved the peripheral circular topology. The line intensitometry performed along such a PML-LMNB1-dashed loop (presented in Figure 9(D^1^) also reveals some chromatin retained there. Unlike these structures, in the small curls of the peripheral circular PML tracts with LMNB1 inside (both often being twisted), such as those shown in Figure 9(E) (asterisks) and 9F (thin double-arrows), the chromatin becomes disjoined from LMNB1 (as exemplified by line intensitometry in Figure 9 (E^1^, E^2^).

In the cells with concentric PML tracts still mostly retained around the nuclear periphery, we also found rare fully colocalised PML-LMNB1 segments, indicating their mutual affinity (Figure 9(F), dashed arrow), or even reverse entrapment of PML into the peripheral cluster of LMNB1 that continued as thick shapeless intranuclear LMNB1 flows (Figure 9(F), arrowhead). We examined the 3D structure of this particular 9F cell in all three channels and the overlay is presented as a video in File S4. The cell was partially flattened by cytospinning. One can find on these 3D images the flows of LMNB1, mostly meridional, scarcely dashed by PML foci, which do not colocalise with the chromatin but, on the contrary, are channeling it and, in the thicker sites, perforate it.

Moreover, in some cells, where PML fibrillar tracts lose their concentric attachment to the peripheral chromatin, it tends to enroll in a few large PML bodies, in close vicinity of the nucleoli (Figure 9(G-I)). The origin and the continuity of these large perinucleolar PML NBs with the peripheral PML tracts detached from the chromatin are seen in Figure 9(G) (red arrow) and Figure 9(H-I) (yellow arrow) – evidenced by the absence of the underlying chromatin. The convolutes of LMNB1 into large perinucleolar PML bodies perforate holes in the chromatin DAPI staining and are frequently continued as a fissure to the nuclear border (red arrows in Figure 9(G-I)). In that, they have mutual features with the nucleolar aggresomes released with centromeres from cell nuclei through autophagic channels and found in the stress response, treated cancer, and congenital laminopathy progeria [67,68]. However, this stage of senescence may also not be terminal, as one or a few nucleoli may remain intact [68]. Note that in the SK-MEL-DOX model the LMNB1 nuclear contour on Figure 9(H) also seems intact or restored, differently from the picture on Figure 9(G).

In general, our observations in this section correspond to those published by Condemine et al. [48,69] using transfected isoforms of PML. As reported, PML NBs assemble in ‘rosettes’ surrounding DNA centromeres or are distributed in tracts bridging two centromeres. The fibrillar isoform PML II targets the inner membrane (lamina) of the nuclear envelope: PML ‘tracts can cross the nucleus and were frequently observed filing lamina gaps at the nuclear envelope’ [48,67]. Furthermore, it can enroll in the PML NB with an absent inner core. PML IV isoform is indispensable for true ALT-APBs, but abundant PML II can both construct fibrillar forms and participate in APBs helping to ‘glue’ telomeric DNA and shelterin proteins; in addition, it can also form various PML aggregates, while PML I and IV isoforms exhibit nucleolar targeting upon stress [70]. Presence of all these PML isoforms was confirmed here by transcriptome analysis (Figure S5). As also recently reviewed [34], the APB encloses two telomere ends, while the giant PML NBs near the nucleoli enclose the amplified satellite centromeric DNA, which was found in the nucleolar aggresomes [68].

In summary, of this section, during the DOX-induced cell senescence process, part of LMNB1 loses the attachment to the peripheral chromatin and flows inside the rotating cell nuclei, while circular thready PML still retains some attachment to the most rigid, likely surface heterochromatin (whose features will be briefly further discussed). When this binding is also lost, the nuclear envelope/LMNB1 support of the nuclear organization appears to be fully broken.

## Discussion

**Figure 10.**
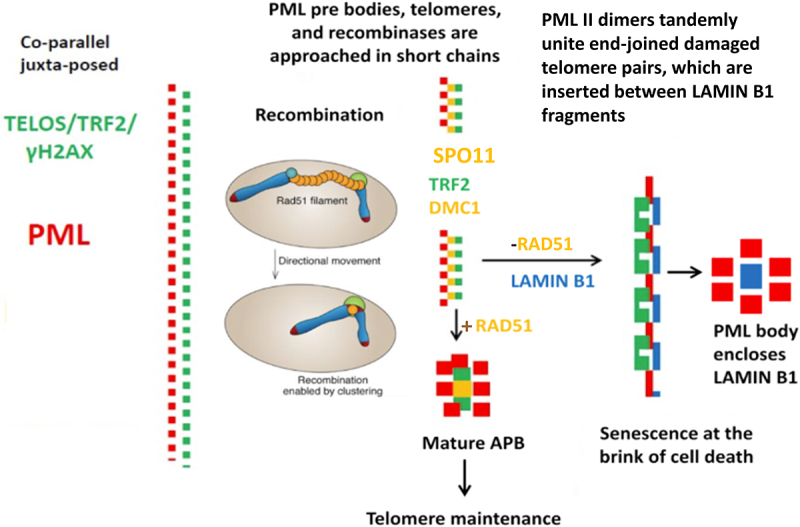
The schematic of the topological relationship between PML-structures, meiotic prophase proteins, the ALT-like process, and nuclear lamin B1 over the course of accelerated cell senescence in DOX-treated cancer cells. The fragment on the mechanism of ALT recombination by RAD51 is reproduced from [18].

A schematic representation of the observed topological relationship between telomeres and meiotic prophase proteins with PML and LMNB1 during DNA repair and misrepair by the ALT-like process in DOX-treated cancer is presented in Figure 10.

Telomeres play a central role in chromosomal and nuclear stability events [71]. In contrast, free chromosome ends with dysfunctional telomeres can trigger chromosomal rearrangements and are involved in evolutionary adaptations [72]. ACS induced in TP53-mutant cancer cells by stress and anticancer drug response also unites two opposite processes: it stops mitotic divisions by MS for one to 3 weeks, yet at the same time the accumulating stressed aneupolyploid cells are continuing DNA rereplication and undergoing RS in the late S-phase ([15] and seen here). The recombinatory ALT-like process seems to be necessary for the restoration of telomeres and mitotic division occurring in a minority of survivors, which can thus provide resistant tumor growth by return to the TERT-driven immortality. At the same time, the ‘double-edged sword’ nature of the ALT-like process needs to be taken into account – its mechanism of action involves break-induced replication (BIR), which can resolve RS [58], but can also cause gene conversion and destabilization of the genome [73,74]. While at the mild DOX dosage we observe an ALT-like process of recombinogenic telomere repair indicated by colocalisation of RAD51/yH2AX/PML foci (ALT itself or BIR), at a sublethal DOX dosage, the things change and the PML II isoform, the only one that can self-multiply in a thread-like form [48], steps on the stage. The conversion of the APB-bodies through PML dimerization stacked by non-repaired telomeric DNA (both TRF2- and γH2AX-positive, as seen from Figure 5) can be explained by the data that PML II also participates in the former, helping to ‘glue’ telomeric DNA and shelterin proteins [70]. What is then observed, may be simplified as follows: The unrepaired telomeres insert segments of PML II in the natural gaps of LNMB1 (described in lymphoma by [43] and, possibly, transfer the unrepaired telomeres to the surface chromatin under the inner nuclear membrane (INM). As a result of this exchange, PML segments are found to be intermitted with LMNB1. Most likely, this can occur by interacting of the hydrophobic hinge segment of the basic Tudor domain of the lamin B receptor (LBR) [75] with the intrinsically disordered exon 7 of the acidic PML II [76]. LBR attached to LMNB1 also spans the INM by eight hydrophobic domains at the C-terminus [75]. In this way, the patches of the damaged telomere DNA may be transferred into the perinuclear space together with envelope limited nuclear sheets (ELCS, see description below) and rotate there.

The heterochromatin, which is more resistant to DOX treatment, longer retaining concentric attachment of PML-tracts may represent the surface ‘epichromatin,’ whose ~1kB domains are located through all chromosomes, particularly at the telomere ends; the domains of epichromatin are bound to the INM through amphiphilic LBR receptor of LMNB1 [75]. Previously, ChIP-seq analysis using a specific antibody for the ternary structure of epichromatin showed that it is enriched with GC-sequences and Alu [77,78]. It is noteworthy that the Gene Ontology analysis revealed the considerable (x18-fold) enrichment of epichromatin with PML-related proteins [77,78] and here we can admit that it may be mostly the ubiquitous PML II isoform. The epichromatin possesses a rigid hydrophobic secondary DNA A-form [79] packed as a row of dense 30-nm globular superstructures [80], different from the AT-rich Lamin-associated domains (LADs). Interestingly, like the concentric PML II tracts interacting with LMNB1 revealed here, epichromatin also participates in the concentric rings of the inner nuclear-envelope-limited chromatin sheets (ELCS) into the perinuclear space being accompanied by LMNB1 [81]. ELCS are long known to be typical for lymphoma and cancer as such [82], enhanced in the stressed polyploid Burkitt’ lymphoma cells [83] and in the differentiating myeloid leukemia [81]. Moreover, ELCS were also found in neural stem cells, and shown to be enriched in telomeres and TRF2 [84]. From the physical point of view, for a soft cavity (soft LMNB1 in our case) larger hard 30-nm globules (epichromatin, in our case) are susceptible to be depleted from the interior of the cavity (ELCS, in our case) as the cavity becomes more crowded [85].

We earlier hypothesized the circulating ELCS to be involved in the search of homology, including sorting of the extrachromosomal circular DNA by autophagy and a secondary repair by HR [68,86]. ELCS that enclose the rows of hydrophobic epichromatin globules rolling along the INM in the perinuclear space can likely well fit for this friction-less movement [79,80].

From a clinical point of view, it is noteworthy that the appearances, described here for two aneuploid tumor cell lines treated by the DNA damaging agent that induced the transient ACS with partial suppression of LMNB1, largely reproduce the observations found in the cells of humans with inherited progeria. In particular, in the laminopathy associated with the Hutchinson-Clifford Syndrome (mutant in Lamin A), the nucleolar aggresomes [67] and the thread-like PML forms colocalised or intermitted with nuclear lamin (also LMNB1) were described in cell culture, after 2 Gy DNA damage [49].

The relationship between ALT-like process and meiotic proteins extends suggested earlier the shared mechanisms of ALT with the meiotic prophase [17–19]. The meiotic nuclease SPO11 and recombinase DMC1, which are usually thought to function in meiosis, were found here interacting with PML to precede the assembly of APB-like bodies, where only RAD51 was localized. In line with that, more recent studies in meiosis show that DMC1 is responsible for the proper assembly of the participants of meiotic recombination and can act independently as a prophase checkpoint upstream to RAD51 [87]. Likewise, homolog pairing in mouse meiosis needs SPO11 before the programmed DNA cleavage [88]. This data may explain the presently observed inclusion of both proteins of the meiotic prophase in short PML chains presetting HR in ALT-like bodies. Moreover, SPO11 generates gaps through the sites of topological stress [89]. The inverted repeats of interstitial telomeres flanking Alu Z-form undergoing torsional stress [90] may also be involved. This data suggest that meiotic genes and pathways contribute to the mitigation of telomeric replication stress, which is inherent in ALT and BIR, as a major component of ACS [4].

It is also important to note that the core meiotic genes, including those revealed in our study of the somatic cancer ALT, are also active in the asexual Entamoeba (Spo11, Hop1, Hop2, Mnd1, Mlh1, Mlh2, Pms1, Dmc1, Msh2, Msh4, Msh5, Msh6, Rad50, Rad51 and Rad52), which suggests a possible ancestral line in the human genome [91]. Therefore, it is highly notable that GO analysis of the epichromatin ChIP-seq data in myeloid leukemia cells, along with 18-fold enrichment with PML (as compared with the general chromatin) also reveals enrichment with modules of the phylopodium membrane (5-fold) and stereocilium tips (2-fold), supporting the ancestral origin of the nuclear envelope mobility associated with PML [78].

The participation of the MOS kinase in endomitosis, which originated in ancient unicellulars and represents an intermediate in the evolution of meiosis from mitosis, and the post-translational stabilization of the meiotic MOS kinase in the DNA-damaged giant somatic tumor cells were analyzed earlier [29–31].

The alternative of these pre-programmed processes may be telomere non-homologous end-joining (NHEJ) or microhomology-mediated ALT-NHEJ, possibly aided by fibrillar PML – to rescue cells by the punctuated event of the ‘genome chaos’ documented in the pedigree of approximately 46% of human cancer genomes [92], which may still be reversible by chromoanagenesis afterward [93]. This NHEJ occurs in the G1/early S-phase and thus may make the non-repaired chromosome ends stick together. The delay in the early S-phase of 8C cells on day 5 after DOX treatment was observed by us in the SK-MEL-28-DOX250 model, which demonstrated a marked difference from the MDA-MB-231-DOX response. This delay may explain the inability of this subfraction to carry out the recombinative RAD51-dependent ALT and its substitution for ALT-NHEJ [94]. The underestimated facet of ‘the evil roots of cancer’ - polyploid giant cancer cells [95], namely the coupled PML NBs dysfunction in epigenetic and post-translational metabolic alterations associated with meiotic elements, becomes better substantiated in our present study. Our data also satisfactorily explains the ‘surprising anti-correlation of LMNB1 with heterochromatin’ enhanced after removing 60–70% of LMNB1 in Burkitt’ lymphoma [43], by liquefaction of LMNB1, which was observed here in the course of the DOX-induced cancer cells senescence.

## Conclusion

The turn of the 21st century started the era dealing with the complexity and the ongoing evolution of the increasingly unstable mammalian and human genomes, which unfortunately drives cancer [96]. The attempts of most researchers in the 20th century to understand cancer through the lens of basic molecular biology and means of biotechnology largely failed due to cancer complexity [97–100]. However, the tremendous efforts to win against cancer began to paradoxically reveal the Biology of Complexity itself as dealing with thermodynamically unstable Adaptive Systems [101,102], with their uncertainty, which is difficult to measure by usual statistics [103], and which is also regulated by molecular crowding [104] at the protein level [105], and by self-emergent processes in confined liquids [85,106,107]. This approach, applied in our analysis of the experimental data to the long-standing enigma of meiotic genes’ role in cancer biology, has revealed to us a novel aspect of meiotic gene involvement in telomere repair, LMNB1 dynamics and heterochromatin organization, through the multifunctional aid of PML structures in epigenetic and post-translational adaptations of aggressive human cancer.

## Supplementary Material

Supplementary.docx

## Data Availability

The raw and processed data of the MDA-MB-231+DOX transcriptomics dataset re-analyzed in this study has been submitted to ArrayExpress and assigned the accession number E-MTAB-16509. An earlier version of this article was uploaded to a preprint server and is currently available at DOI:10.20944/preprints202401.0111.v2.
