# Supplementary material for: Repair and misrepair of telomeric DNA in dynamic interactions with PML nuclear bodies and lamin B1 in doxorubicin-treated cancer cells
Source: Nucleus. 2026 Jul 23;17(1):2688655. doi: 10.1080/19491034.2026.2688655 (PMC13398099; doi:10.1080/19491034.2026.2688655)
Supplement: Supplementary.docx [file KNCL_A_2688655_SM0372.docx]

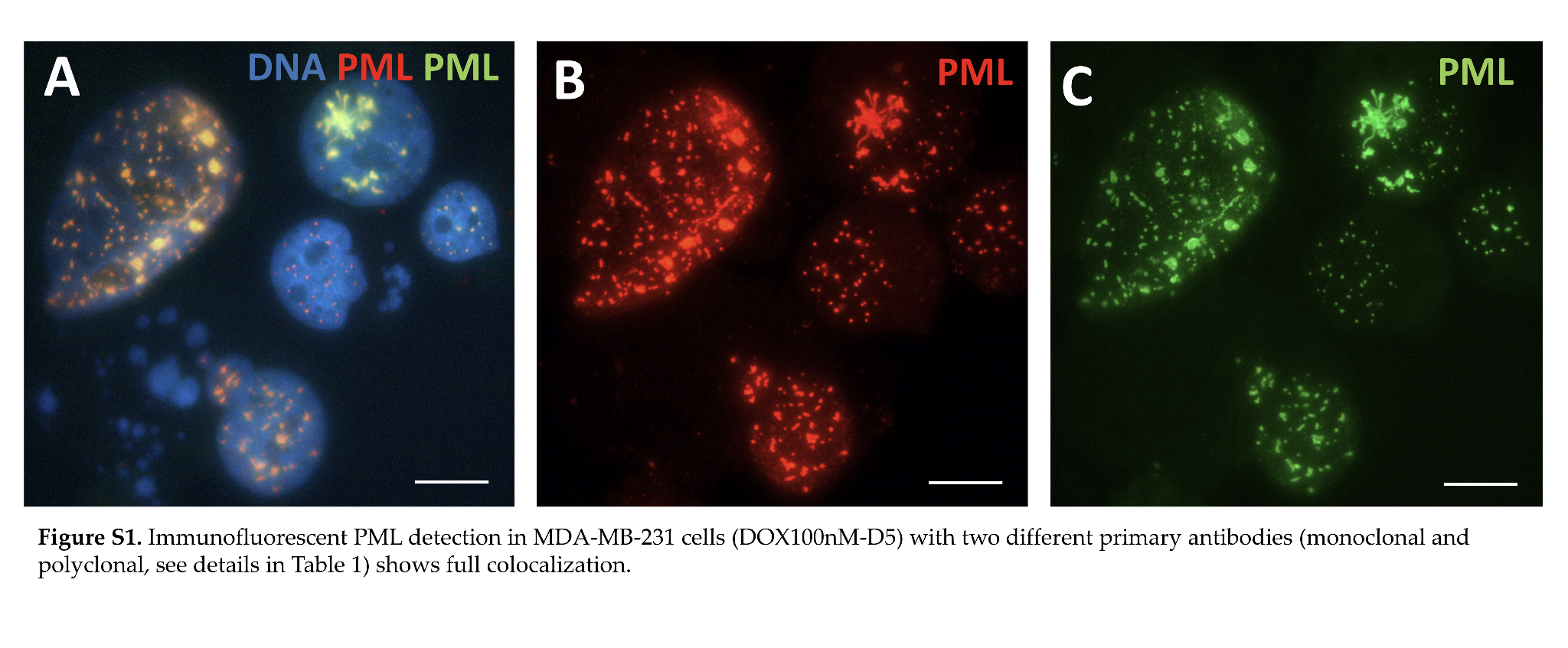


Figure S1. Immunofluorescent PML detection in MDA-MB-231 cells (DOX100nm-D5) with two different primary antibodies (monoclonal and polyclonal, see details in Table 1) shows full colocalization.


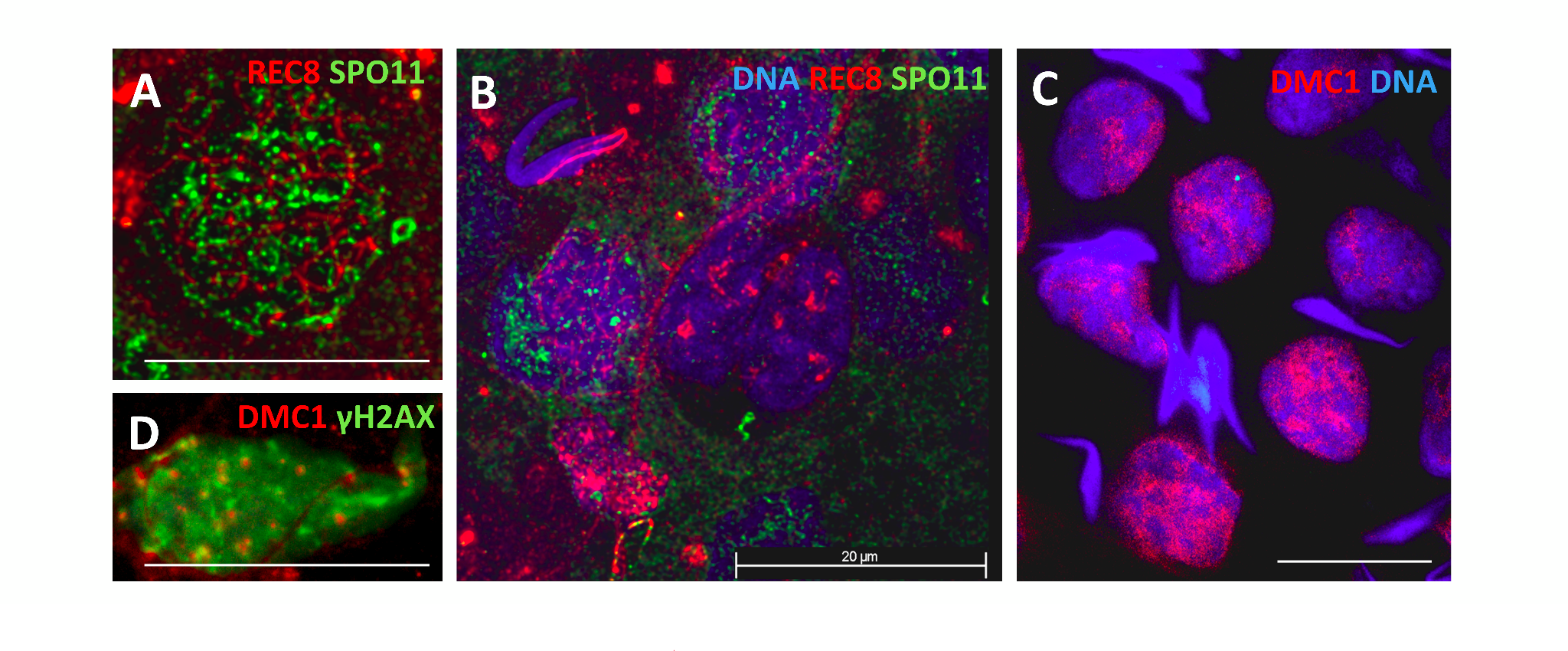


Figure S2. Verification of the meiotic protein antibodies in rat testis: (A-C) Synaptonemal complexes of rat spermatocytes I are positive for SPO11, DMC1 and REC8. (D) A round spermatid, beginning elongation, contains remnant $\gamma$H2AX-positive DNA double-strand breaks colocalised with DMC1. (B, C) methanol fixation. Bars=20$\mu$m.

File S3. Differentially expressed genes (DEGs) between different timepoints of a time-series MDA-MB-231+DOX RNA-seq dataset.

<https://www.dropbox.com/scl/fi/b5wtmxxyj5tdde0ibt3ue/Supplementary-File-S3.xlsx?rlkey=4rao6mdmqgrw4vfi296ro8xe9&st=aw61md74&dl=0>

File S4. Video of confocal images of 3D structure of a cell (SK-MEL-28 on D5 after DOX, from Fig.9F) in all three channels and the overlay, showing PML fibrillar structures in their topological relationship with Lamin B1 (LMNB1) and chromatin.

<https://www.dropbox.com/scl/fi/qa28kxhu7jqg2nd0z6ake/Supplementary-video-S2.mp4?rlkey=k1pc8a7r14r10osssmv0pp2pu&st=6gktcthv&dl=0>


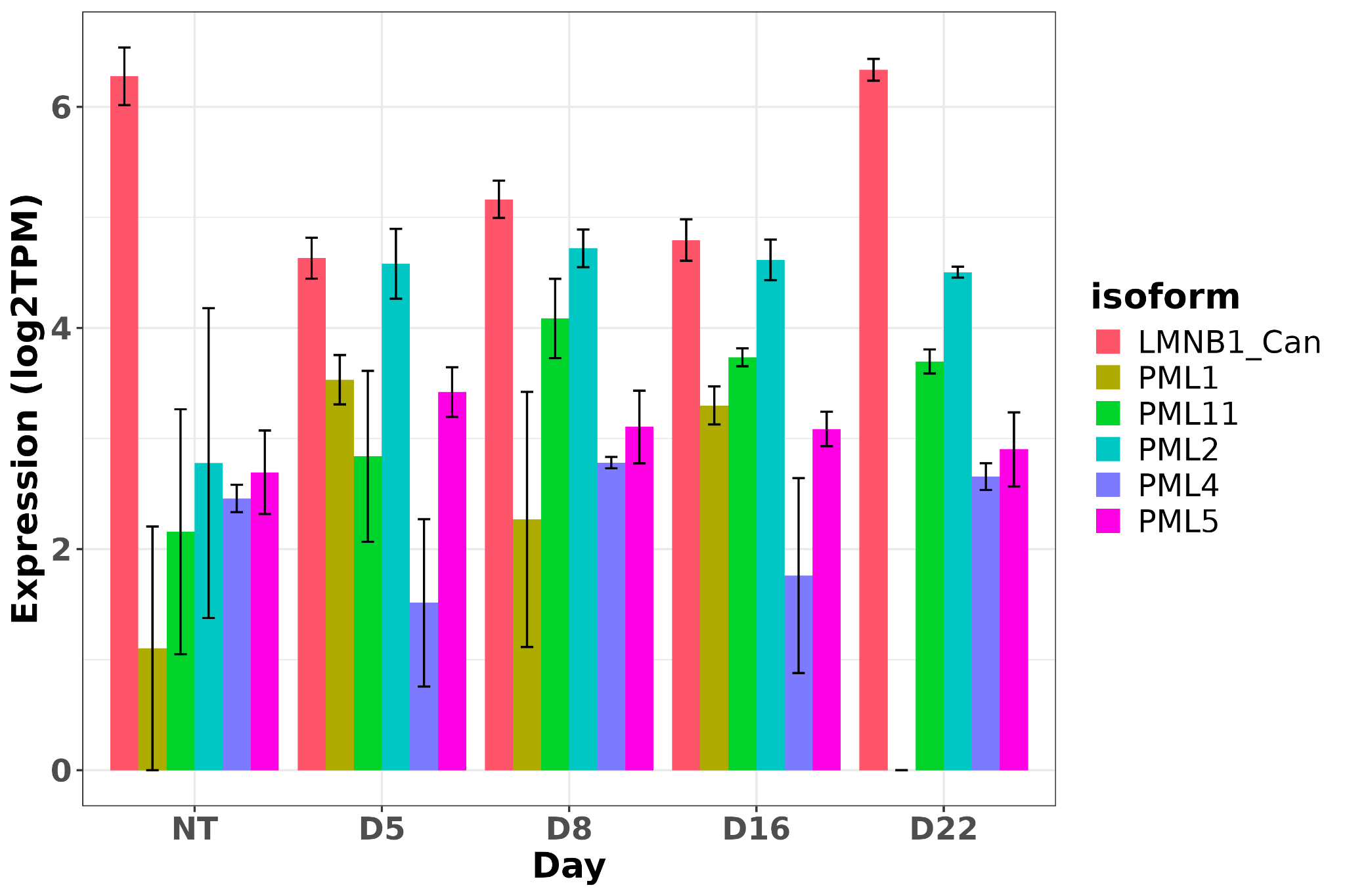


Figure S5. The barplot visualization of LMNB1 canonical isoform and various PML isoform transcript expression over different time-points in the MDA-MB-231+DOX dataset, showing robust LMNB1 expression, as well as the presence of PML1, PML2, PML4, PML5 and PML11 isoforms.
